# Inclusion/Exclusion Behaviors of Small Molecules during Crystallization of Polymers in Miscible PLLA/TAIC Blend

**DOI:** 10.3390/polym14132737

**Published:** 2022-07-04

**Authors:** Lu Yin, Jiayao Wang, Taotao Lin, Jichun You

**Affiliations:** Key Laboratory of Organosilicon Chemistry and Material Technology, Ministry of Education, College of Material, Chemistry and Chemical Engineering, Hangzhou Normal University, No. 2318, Yuhangtang Rd, Hangzhou 311121, China; yinlu9898@163.com (L.Y.); jiayao-wang@outlook.com (J.W.); lintaotao1417@163.com (T.L.)

**Keywords:** localization, SAXS, PLLA, melt crystallization, cold crystallization

## Abstract

In this work, PLLA/TAIC has been taken as a model system to investigate the inclusion and exclusion of small molecules during the crystallization of polymers in their miscible blend. Our results indicate that it is the growth rate and diameter of PLLA spherulites that dominate the localization of TAIC. On the one hand, crystallization temperature plays an important role. Crystallization at higher temperature corresponds to higher growth rates and a greater diameter of PLLA spherulites. The former improves the ability of PLLA crystals to trap TAIC while the latter leads to a lower volume fraction of space among neighboring PLLA spherulites. The combination of the two contributes to the enhanced inclusion behaviors. On the other hand, when compared to melt crystallization, cold crystallization results in much smaller spherulites (from higher nucleation density) and sufficient space among spherulites, which accounts for the enrichment of TAIC in interspherulitic regions and for its enhanced exclusion. In the adopted polymer–small molecule blend, TAIC can enrich in interspherulitic regions even in its miscible blend with PLLA, which can be attributed to its stronger diffusion ability.

## 1. Introduction

Polymeric materials have been widely used in many fields, in most of which they are blended with components including other polymers or additives [1,2,3,4]. Depending upon the miscibility between components, there are immiscible blends and miscible blends [5,6]. The scenario becomes more complicated when one component is crystalline. In miscible polymer blends containing at least one crystalline component (known as component A), the amorphous component or lower melting temperature component (known as component B) can enrich in interlamellar, interfibrillar (known as interlamellae stack), or interspherulitic regions [7,8,9,10]. This is the so-called phase segregation in miscible polymer blends containing crystalline components, which is completely different from phase separation arising from the higher magnitudes of Flory–Huggins interaction parameters. Much effort has been made to investigate the localization of component B. It has been indicated that both the diffusion coefficient (*D*) of B and the crystal growth rate (*G*) of A play important roles in determining the localization of component B. In the case of higher *G* and lower *D*, component B is trapped by the crystal lamellae of A, leading to phase segregation in interlamellar regions, while extremely high *D* and low *G* contribute to the localization of B among spherulites of A (interspherulitic regions). Of course, the enrichment of the amorphous component in interfibrillar regions occurs when the spherulites of A grow slowly and component B exhibits relatively strong diffusion ability. The evolution from an interspherulitic regime to an interlamellar regime is known as inclusion while the opposite phenomenon is known as exclusion. The inclusion and exclusion behaviors of component B are dominated by the ratio between its diffusion coefficient and the crystal growth rate of A, both of which are under the control of the blend composition, the adopted crystallization temperature, and the molecular weight of polymers [11,12]. For instance, Ye et al. achieved precise interlamellar/interfibrillar localization in poly(vinylidene fluoride)/poly(1,4-butylene succinate) (i.e., PVDF/PBSU) blends with different compositions. Similar volume fractions of account for the interlamellar structures of these blends. When PVDF acts as the minority phase, it locates between PBSU crystal stacks, which is the reason for interfibrillar structures [13]. In the studies of Lin, the effect of poly(methyl methacrylate) (PMMA) molecular weight on its localization during the crystallization of PVDF in their miscible blend were investigated in detail by the combination of small-angle X-ray scattering (SAXS) and differential scanning calorimetry (DSC). The results indicate that the higher molecular weight of PMMA corresponds to higher viscosity and reduced crystal growth rate (*G*) of PVDF, accounting for the enhanced exclusion behaviors and bigger pores after removal of PMMA [14].

There have been some reports concerning the inclusion and exclusion behaviors of component B during the crystallization of component A. Most of these, however, focused on polymer–polymer blends [15,16]. It is important to pay attention to the blending of polymers and small molecules due to the following issues: Firstly, in industry, polymers have always been blended with small molecules to optimize processing conditions and enhance aspects of performance including flexibility, ductility, workability, and extensibility [17]. For instance, several kinds of plasticizers for nylon 66 (or nylon 6) were studied by Narkis and his co-workers [18]. In the research of Baiardo et al., poly(ethylene glycols) (PEGs) and acetyl tri-n-butyl citrate were used to plasticize poly(L-lactic acid) (PLLA) [19]. Based on the special intermolecular interactions between polymers (nylon or PLLA) and plasticizers, the plasticized blends exhibited excellent miscibility. We can therefore understand this to be a typical miscible polymer–small molecule blend system containing a crystalline component. Secondly, *D* and *G* always exhibit a strong simultaneous dependence on treatment conditions in polymer–polymer blends. For example, when isothermal crystallization temperature is adjusted, there are variations in *D* and *G* at the same time, leading to complex enrichment or segregation behaviors [20,21,22,23,24,25]. In the polymer–small molecule blend, however, the diffusion coefficient of the latter exhibits a much higher magnitude. The small molecule temperature dependence of is not as sensitive as that of polymer. The polymer growth rate depends crucially on the isothermal crystallization temperature. As a result, it is a simple task to tailor the inclusion and exclusion behaviors of small molecules during the crystallization of polymers according to the different temperature dependences of *D* and *G*. Finally, the investigation of polymer–small molecule blend systems has significance for some special applications, e.g., the enhanced bioavailability of drugs. It is well known that amorphous drugs exhibit higher dissolution and solubility in water compared to crystalline forms. The confinement effect of their localization in interlamellar, interfibrillar or interspherulitic regions can inhibit their crystallization and enhance bioavailability [26].

In this work, therefore, a blend of poly(L-lactic acid) (PLLA) and triallyl isocyanate (TAIC) was taken as a model system for this purpose. After the validation of miscibility between them, the localization of TAIC upon isothermal melt crystallization and cold crystallization of PLLA at various temperatures was examined by a combination of SAXS and DSC. The inclusion and exclusion behaviors of TAIC during the crystallization as well as their temperature dependences were then investigated and compared with the results of polymer–polymer blends [27].

## 2. Experimental Section

### 2.1. Materials

Poly L-lactide (PLLA, 3001D, *M*_n_ = 8.9 × 104 g/mol, *M*_w_/*M*_n_ = 2.0) was supplied by Nature Works, Blair, NE, USA. Triallyl isocyanurate (TAIC) was provided by Sinopharm Chemical Reagent Co., Ltd., Shanghai, China. Methanol (CH_3_OH, AR) was purchased from Shanghai Lingfeng Chemical Reagent Co., Ltd., Shanghai, China.

### 2.2. Sample Preparation

Preparation of PLLA/TAIC blends: Prior to melt blending, PLLA was vacuum-dried at 80 ºC for at least 4 h to remove any moisture, which is an efficient way to avoid hydrolytic degradation of PLLA during the melting process. PLLA and TAIC blends were prepared by melt blending in a Haake internal mixer (Vreden, Germany). The preparation process was as follows: The PLLA was mixed with TAIC (the mass ratios of PLLA/TAIC were 60/40, 70/30, and 80/20) at 190 °C in the batch mixer (Haake Polylab QC with blade rotor and capacity of 50 g) with a twin screw at an initial rotation speed of 20 rpm (2 min) subsequently raised to 50 rpm (10 min). Samples with PLLA weight fractions of 60%, 70%, and 80% were prepared by hot press at 200 °C under 10 MPa pressure for 3 min to produce a film with a thickness of 300 μm. The prepared films were then cooled to room temperature by a flat vulcanizer (MZ-3012) and stored in a refrigerator (−20 °C).

Melt crystallization: An appropriate amount of the blend was placed on a Linkam LTS 350 hot stage at 200 °C for 5 min so that it melted completely. Then, the specimen was cooled down to a certain temperature by flowing ethanol reagent through the circulation pipe at a rate of 100 °C/min. For sufficient crystallization, the specimen was kept at the specified temperature for 2 h.

Cold crystallization: An appropriate amount of the blend (previously stored in a refrigerator) was placed on a preheated hot stage at a certain temperature. To achieve sufficient crystallization, the specimen was kept at this temperature for 30 min.

Preparation of PLLA porous material: For the blended specimens, upon crystallization, methanol was used as a selective solvent to etch TAIC by immersing specimens in methanol at room temperature for 24 h. Then, the film was solidified in a refrigerator at −20 °C using water instead of solvent, and then lyophilized in a lyophilizer (−100 °C).

### 2.3. Characterization

#### 2.3.1. Morphology Structure

SEM (FE-SEM, Hitachi S-4800, Tokyo, Japan) was used to observe the cross-section morphology of pure PLLA, PLLA/TAIC blends, and PLLA/TAIC porous material at an accelerating voltage of 5.0 kV. The specimens were fractured by immersing in liquid nitrogen for 5 min and then spur-coated with gold. TEM (Hitachi HT-7700, Tokyo, Japan) was employed to observe the morphologies of PLLA/TACI sliced sections (~80 nm thickness) after staining with ruthenium tetroxide for 4 h.

#### 2.3.2. Thermal Analysis

The glass transition temperature and the melting point of PLLA/TAIC blends were measured by differential scanning calorimetry (DSC, Q2000, TA Instruments, New Castle, PA, USA). Under nitrogen atmosphere, the sample (3–5 mg) was heated from 0 °C to 200 °C at a rate of 10 °C/min, the thermal history was eliminated at 200 °C for 5 min, and temperature was then lowered from 200 °C to 0 °C at the same rate per minute as before. To determine the crystallinity of the completely crystallized PLLA/TAIC blends, the sample was heated from 0 °C to 200 °C at a rate of 10 °C/min. The DSC curve of the recording sample was then recorded.

#### 2.3.3. Crystallization Kinetics

The crystallization kinetics of PLLA/TAIC blends were examined by differential scanning calorimetry (DSC, Q2000, TA Instruments, New Castle, PA, USA). During the isothermal crystallization test, an appropriate amount of blend sample was taken and heated from 0 °C to 200 °C at a heating rate of 10 °C/min and kept at 200 °C for 5 min in a nitrogen atmosphere, by which the thermal history was eliminated. The temperature was then lowered to the crystallization temperature *T*_c_ at a rate of 100 °C/min and maintained until crystallization was complete. The DSC curve of the sample was then recorded.

#### 2.3.4. Crystal Lamellar Structure

The lamellar structure of the blends was examined by small-angle X-ray scattering (SAXS, Shanghai Synchrotron Radiation Facility, Shanghai, China) of beamline BL16B1. The X-ray wavelength was 0.124 nm and the sample-detector distance was1980 mm.

#### 2.3.5. Crystal Morphology

The morphologies of PLLA/TAIC blends were imaged with a polarizing optical microscope (POM, Olympus BX51, Tokyo, Japan) equipped with a digital camera. All PLLA/TAIC samples were sandwiched between two plates of glass with the temperature controlled by a Linkam LTS 350 hot stage. The temperature was heated to 200 °C for 5 min to eliminate the thermal history, followed by cooling the crystallization temperature at a rate of 100 °C/min and crystallized completely.

## 3. Results and Discussion

**Figure 1:**
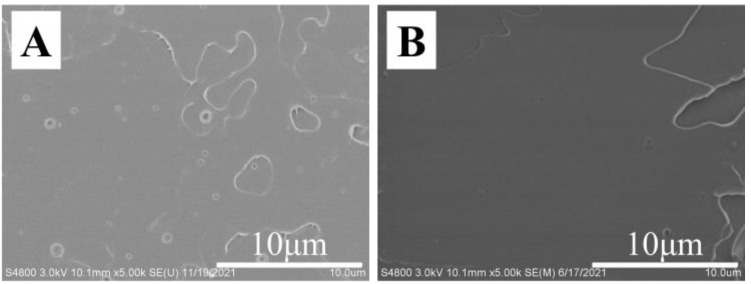

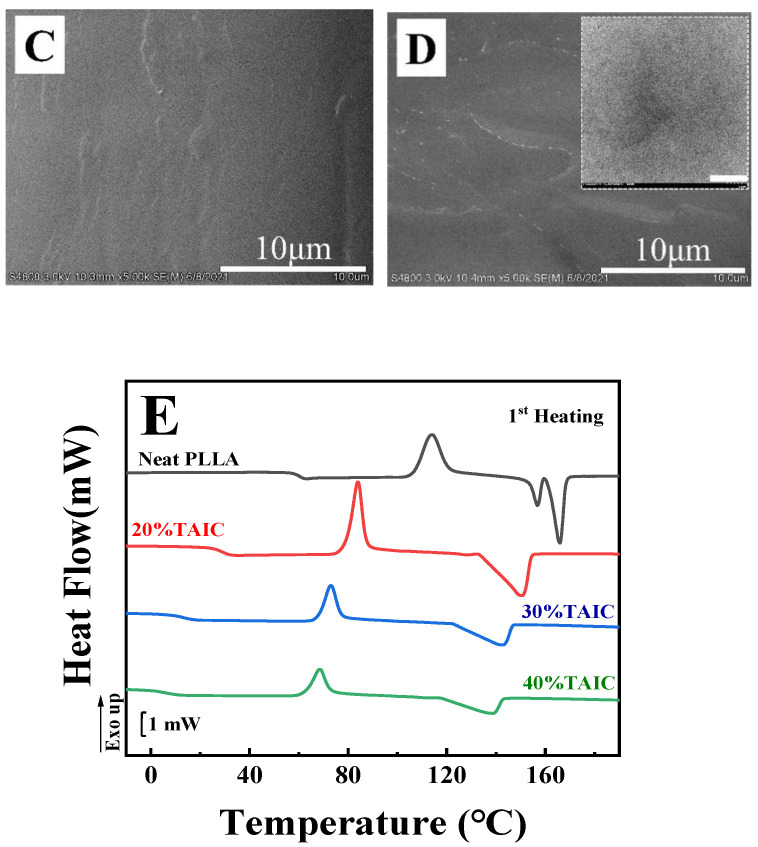
SEM images (**A**–**D**) and DSC curves (**E**) of cross section of PLLA/TAIC blend films with various compositions: (**A**)—neat PLLA, (**B**)—PLLA/TAIC (80/20), (**C**)—PLLA/TAIC (70/30), (**D**)— PLLA/TAIC (60/40). Inset in (**D**) shows the TEM image of the corresponding specimen in which the scale bar is 500 nm.

First of all, the thermodynamic miscibility between PLLA and TAIC was assessed by means of SEM and DSC. In the blend with various compositions of PLLA and TAIC, the melt-blended specimens were obtained by hot pressing. In the SEM images of the fracture surfaces (Figure 1A–D), there is no evidence of any aggregation of TAIC even when the weight fraction of TAIC reaches 40% (Figure 1A–D). The homogeneous distribution of TAIC in PLLA indicates excellent miscibility between them. In Figure 1E, showing the DSC curves of PLLA/TAIC blends, attention should be paid to the following issues: Firstly, the glass transition temperature (*T*_g_) of neat PLLA is located at 60.2 °C. Upon blending with TAIC, the value *T*_g_ of the blend decreases notably. In the green curve (40% TAIC), it is located at 6.9 °C. For all specimens, there is only one glass transition temperature. Secondly, two melting peaks at 156.8 °C and 165.9 °C in neat PLLA are obvious. The main melting temperature (*T*_m_) moves in a lower direction upon blending with TAIC. In the specimens with 20%, 30% and 40% TAIC, it decreases to 150.5 °C, 142.8 °C and 138.8 °C, respectively. The remarkable decrease in melting temperature (*T*_m_) suggests that TAIC causes an obvious effect on the crystallization of PLLA. This is the well-known “*T*_m_ depression effect” reported by Nishi and Wang [28]. This effect has always been observed in miscible polymer blends and has thus been regarded as a method to assess the miscibility of adopted blends [13,14,15,27]. Obviously, this phenomenon can be attributed to the depression of crystallization of PLLA due to the existence of TAIC and subsequent melting behaviors during heating. The evolutions of *T*_g_ and *T*_m_ shown in Figure 1E make it clear that PLLA and TAIC are thermodynamically miscible blends. According to our analysis of the SEM and DSC results, the miscibility between PLLA and TAIC is confirmed, which agrees with results previously reported in the literature [29,30,31,32]. This will be the basis for the following investigation of inclusion and exclusion behaviors of TAIC during the crystallization of PLLA. To observe the inclusion and exclusion behavior of TAIC more clearly, the blend with composition of 60/40 (PLLA/TAIC) is adopted for the following sections.

**Figure 2:**
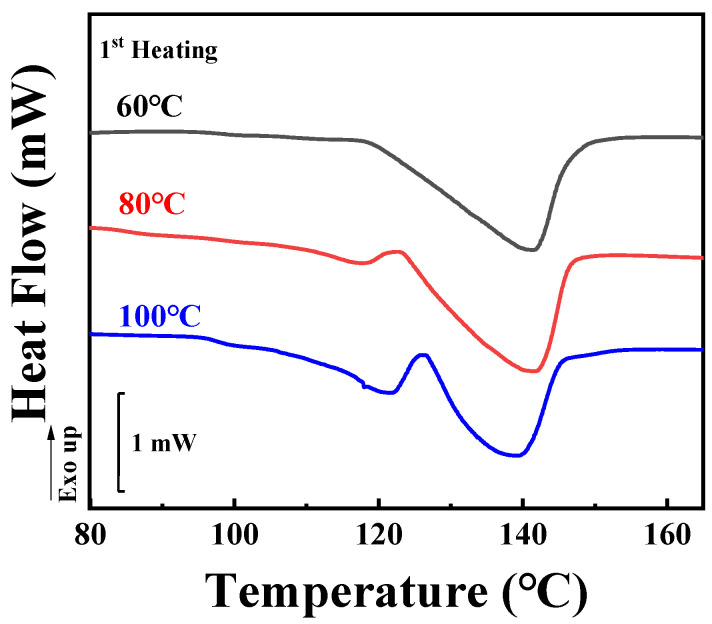
DSC heating curves of the PLLA/TAIC (60/40) blend after complete melt crystallization at the indicated temperatures.

To assess the inclusion or exclusion behaviors of TAIC during the crystallization of PLLA, the crystallinity ratio between SAXS and DSC, as introduced by Stühn, was considered [33]. In this work, the localization of TAIC during the crystallization of PLLA was investigated for both melt crystallization and cold crystallization. In the former, the specimens were heated to 200 °C and maintained at this temperature for 5 min to eliminate the effect of thermal history. Temperature was then maintained for 2 h at various temperatures sufficient for isothermal melt crystallization. For this work, three crystallization temperatures of 60 °C, 80 °C, and 100 °C were adopted according to DSC (Figure 1 and Figure 2) and POM results (shown in the following sections). After preparation, DSC was employed to measure the crystallinity of the blend. As shown in Figure 2, there is only one endothermic peak located at 141.4 °C, representing the melting of PLLA crystals formed during isothermal crystallization at 60 °C. In the red (80 °C) and blue (100 °C) curves, another peak appears in addition to the peak shown in the black curve. This peak becomes more obvious in the case of isothermal crystallization at the highest temperature (100 °C). It can be assigned to the disorder-to-order transformation of α’ to α crystals of PLLA which has been discussed by Zhang and co-workers [34]. Based on the DSC heating curves shown in Figure 2, it is a simple matter to calculate the overall crystallinity in PLLA/TAIC blend according to Equation (1).

(1) $$X_{w}=\frac{\Delta H_{m}}{\Delta H_{m}^{\theta }}\times 100\%$$

where $\Delta H_{m}$ and $\Delta H_{m}^{\theta }$ are the melting enthalpy and standard melting enthalpy of PLLA, respectively. For this work, 93.7 J/g was taken as the value of the latter [35]. It is noteworthy that the overall crystallinity is defined as the weight fraction of crystallized PLLA over the weight of whole blend (not the weight of PLLA). The values of crystallinity are listed in Table 1 (*X*_2_).

**Figure 3:**
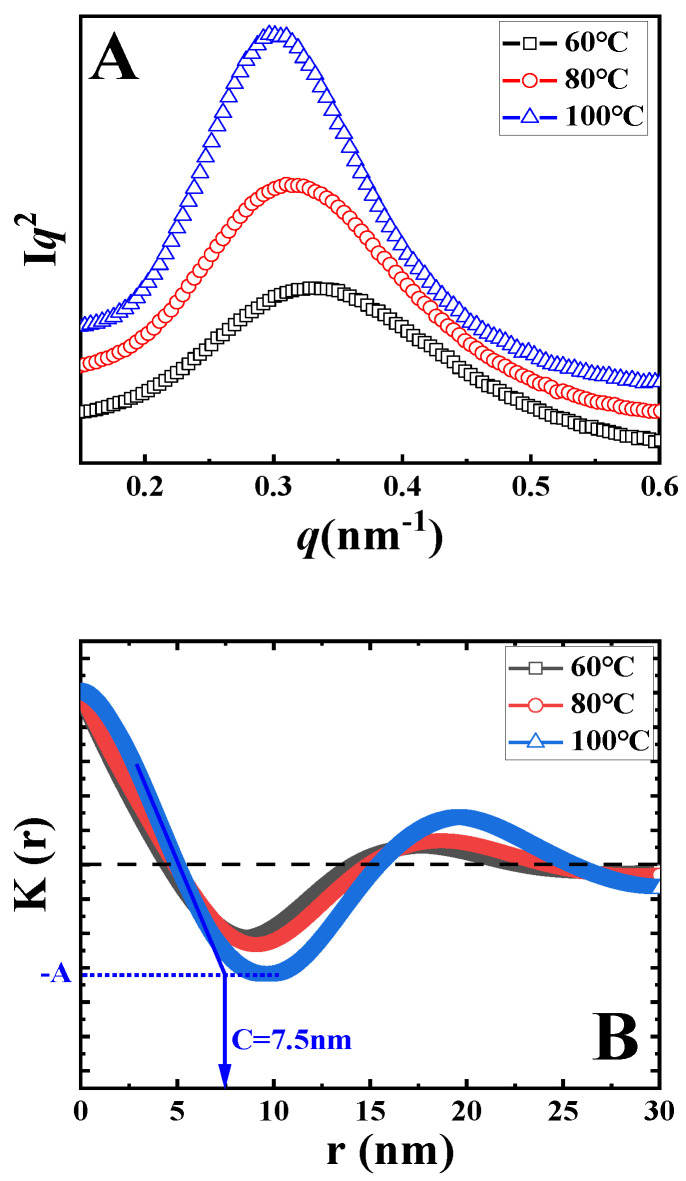
The Lorentz-corrected SAXS profiles (**A**) and one-dimension correlation functions (**B**) of the PLLA/TAIC (60/40) blend after melt crystallization at the indicated temperatures.

The Lorentz-corrected SAXS curves of samples upon isothermal melt crystallization at the indicated temperatures are shown in Figure 3A. In all curves, there is only one scattering peak. In the black curve, the peak is at 0.33 nm^−1^, corresponding to the long period of 18.9 nm. With the increase in crystallization temperature, the peak shifts to the left, accounting for the higher magnitude of the long period. In accordance to the method discussed above, the long periods after isothermal crystallization at various temperatures were identified, as listed in Table 1 (*L*). To determine the thickness of crystal lamellae (*d*) from SAXS data, one-dimensional correlation functions *K(z)* were calculated (i.e., the Fourier transformation of scattering curve) based on Equation (2).

(2) $$K\left(z\right)=[\int_{0}^{\infty }q^{2}I\left(q\right)cos\left(qz\right)dq]/2\pi$$

where *q* and *I* represent the scattering vector and scattering intensity, respectively [33]. The resultant correlation functions *K(z)* are shown in Figure 3B. The identification of lamellae thickness can now be introduced by taking the blue curve as an example. In Figure 3B, the lowest magnitude in *K(z)* has been defined as *−A*. The value along the r-axis of the crossover between the extrapolation of the initial slope for *K(z)* and the line *K(z)* = *−A* represents the thickness of crystal lamellae or the amorphous layer, depending on the value of crystallinity. In Figure 3B, the average thickness is defined as the crystal lamellae thickness because the crystallinities of PLLA are lower than 50%. In this way, the values of crystal lamellae thickness for all specimens were calculated, as listed in Table 1.

To assess the localization of TAIC during the crystallization of PLLA, we paid particular attention to the ratio of *X*_1_/*X*_2_ (Table 1) introduced by Stühn and his co-workers using a combination of SAXS and DSC [33]. Firstly, the internal crystallinity (*X*_1_) was defined as the ratio between *d* and *L*, where *d* and *L* represent the thickness of crystal lamellae and the long periods of the blend respectively. Both of these can be obtained from the SAXS data or from one-dimensional correlation functions (Figure 3). Secondly, the overall crystallinity of the PLLA/TAIC blend (*X*_2_) from DSC was calculated based on the enthalpy values. The magnitude of the crystallinity ratio of *X*_1_/*X*_2_ could then be easily determined. This ratio can act as an effective parameter to describe the extent of TAIC exclusion. When all TAIC locates in the interlamellar regions of PLLA crystals, *X*_1_ and *X*_2_ should exhibit the same magnitude, resulting in a ratio of 1. In other words, the lower value of the ratio corresponds to the enhanced inclusion (i.e., reduced exclusion) of TAIC. For the specimen of neat PLLA upon melt crystallization at 100 °C, the values of *L*, *d*, *AM*, *X*_1_ and *X*_2_ are 17.7 nm, 7.3 nm, 10.4 nm, 41.2% and 42.3% respectively, contributing to the magnitude of *X*_1_/*X*_2_ close to 1. Obviously, AM exhibits a higher magnitude in the PLLA/TAIC blend (13.7 nm) compared to neat PLLA (10.4 nm). This result indicates that TAIC is trapped in the interlamellar regions during the crystallization of PLLA, suggesting miscibility between them (Figure 1). As shown in Table 1, this ratio is 1.31 in the case of isothermal crystallization at 60 °C. With increasing crystallization temperature, it decreases to 1.27 and 1.24 at 80 °C and 100 °C, respectively. These results indicate that more TAIC is trapped in interlamellar regions of PLLA crystals at higher isothermal crystallization temperatures.

**Figure 4:**
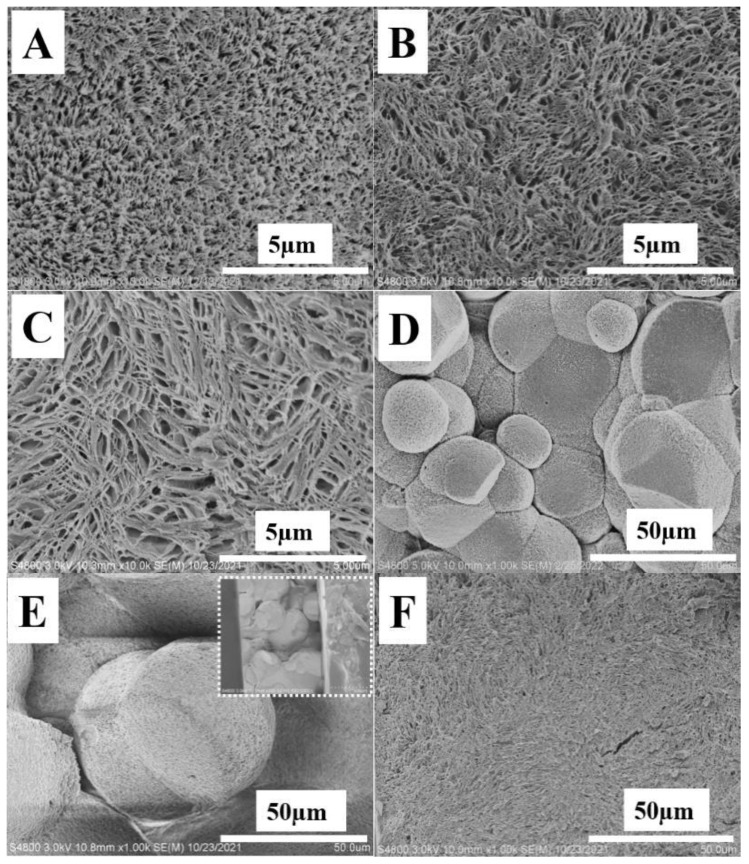
SEM images of PLLA/TAIC (60/40) blend upon etching with methanol on the specimen after complete melt crystallization at 60 °C (**A**,**D**), 80 °C (**B**,**E**), and 100 °C (**C**,**F**).

The bi-continuous structures including the crystal framework and the amorphous components of polymer blend systems have been validated in our previous work [13,14,27,36,37,38,39]. Based on this model, a novel strategy has been developed to fabricate porous structures upon etching with selective solvent. The resulting porous structures depend crucially on the localization of amorphous or lower melting temperature components. Therefore, these porous structures can be employed to identify the location of TAIC in the PLLA crystal framework. As shown in Figure 4, porous structures can be obtained upon etching TAIC with the suitable solvent of methanol, indicating the excellent bi-continuous structures of the blend. Their size can be measured in tens of nanometers at 60 °C (Figure 4A), rising to above 100 nm at 80 °C (Figure 4B). The isothermal crystallization at 100 °C produces porous structures with a size of ~300 nm (Figure 4C). It is worth noting that the structures on fracture surfaces under lower magnifications (Figure 4D–F) also exhibit obvious dependence on isothermal crystallization temperatures. In Figure 4D,E, there can be seen some polyhedral boundaries, resulting from the spherulites impingement [40]. Their occurrence suggests the poor connectivity of spherulites during fracture in liquid nitrogen, and further suggests the enrichment of TAIC in interspherulitic regions [27]. The size of spherulites at 60 °C ranges from 15 µm to 40 µm (Figure 4D). This value increases significantly with an increased crystallization temperature to 80 °C (Figure 4E). At 100 °C (Figure 4F), the spherulites are too big to observe at the magnification used. The increase in spherulite size can be attributed to lower nucleation density at higher temperature. Based on SEM images with various magnifications, we can draw the conclusion that isothermal crystallization at lower temperature enhances the exclusion behaviors of TAIC, producing the favorite enrichment of it in interspherulitic regions (Figure 4D). This is the reason for the spherulites impingement (Figure 4D) and smaller pores (Figure 4A). The increase in crystallization temperature is beneficial for the inclusion of TAIC, accounting for its migration to the amorphous regions in PLLA spherulites for the bigger pores shown in Figure 4C.

**Figure 5:**
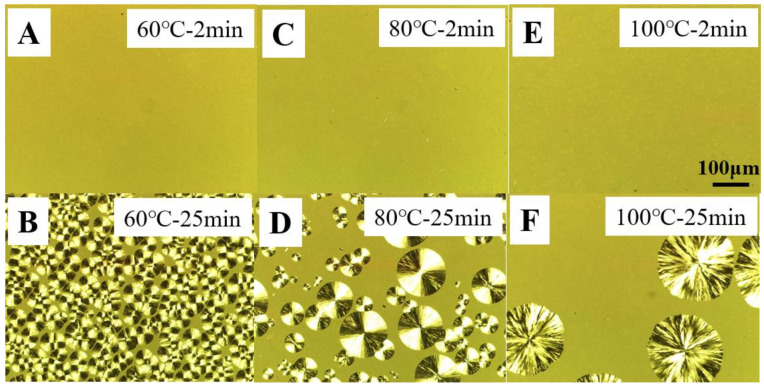
POM images of the PLLA/TAIC (60/40) blend upon isothermal melt crystallization at 60 °C (**A**,**B**), 80 °C (**C**,**D**), and 100 °C (**E**,**F**) for the indicated time periods.

To clarify the reason for different localizations of TAIC, we pay attention to the diffusion coefficient of TAIC (*D*) and to the growth rate of PLLA spherulites (*G*). Compared to polymer, TAIC exhibits a much higher magnitude of *D*. The localization of TAIC, therefore, is mainly dominated by *G*. After being melted at 200 °C for 5 min, the blend was cooled to certain temperatures, then observed by means of POM. In the POM images shown in Figure 5, we can see there are no structures after 2 min (Figure 5A,C,E). After crystallization for 25 min, there are so many spherulites (Figure 5B) that their sizes exhibit lower magnitudes (~23 µm). In Figure 5D,F, the number of spherulites decreases while their size increases significantly. At 100 °C, the size of spherulites reaches ~200 µm. The average growth rates of PLLA spherulites can now be calculated easily. They are 1.0 µm/min, 2.3 µm/min and 8.0 µm/min for temperatures of 60 °C, 80 °C and 100 °C, respectively. Obviously, higher crystallization temperature corresponds to a higher magnitude of growth rate. At 60 °C, the growth rate of PLLA spherulites exhibits a lower magnitude. It is difficult for the spherulite to trap TAIC at this temperature. In addition, the higher nucleation density results in a higher volume fraction of interspherulitic space (Figure 5B). The failure of inclusion and of the extensive space among spherulites both contribute to the localization of TAIC in interspherulitic regions. Upon etching with methanol, TAIC was removed, producing poor connectivity of these spherulites. The polyhedral boundaries from the spherulites impingement which can be observed in Figure 4D is caused by poor connectivity resulting from reduced mechanical performance in these regions. This is the reason for the enhanced exclusion behaviors. This result is in agreement with the higher value of *X*_1_/*X*_2_ (1.31) shown in Table 1. At a crystallization temperature of 100 °C, the higher growth rate of PLLA spherulites makes it possible to trap TAIC. The lower nucleation density (Figure 5F) reduces the volume fraction of interspherulitic space. The combination of them contributes to the enhanced inclusion behaviors and lower value of *X*_1_/*X*_2_ (1.24) in Table 1.

**Figure 6:**
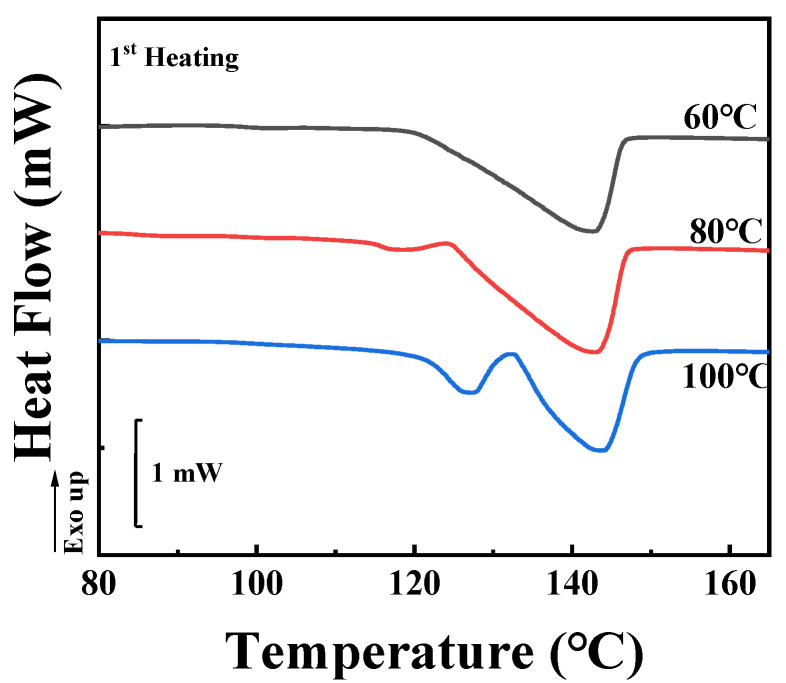
DSC heating curves of the PLLA/TAIC (60/40) blend upon complete cold crystallization at the indicated temperatures.

In the following section, we will focus on the results of cold crystallization, in which specimens previously stored in a refrigerator were heated to certain temperatures (60 °C, 80 °C, and 100 °C) and maintained at these temperatures for a period sufficient for crystallization to occur (30 min). Figure 6 shows the DSC first-heating curves of the specimens. The melting peaks are located at similar temperatures to the corresponding results for melt crystallization (Figure 2). It should be noticed that the axes of heat flow in Figure 2 and Figure 6 are on the same scale. Based on the enthalpies shown in Figure 6, the overall crystallinities from DSC (*X*_2_) were calculated according to Equation (1). Their values are listed in Table 2. For temperatures of 60 °C, 80 °C and 100 °C, they are 21.5%, 22.1% and 23.0%, respectively. These values are much lower than the results from melt crystallization (Figure 2 and Table 1). The reason for this will be discussed in the following sections.

**Figure 7:**
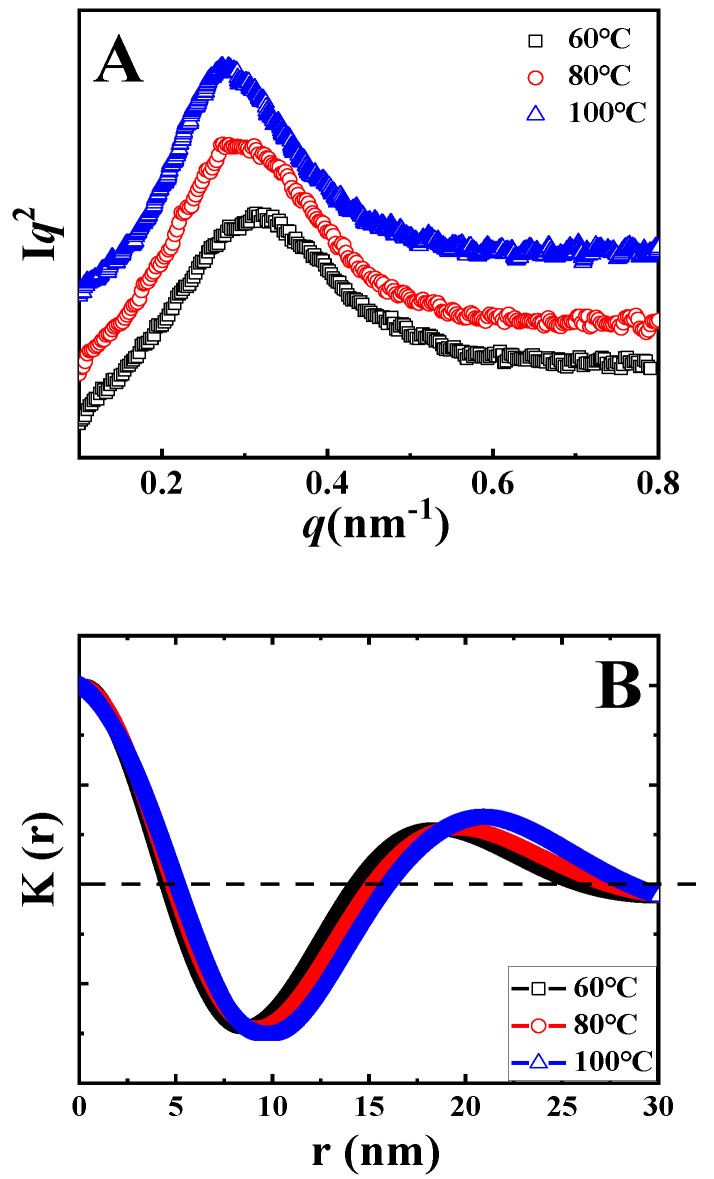
The Lorentz-corrected SAXS profiles (**A**) and one-dimensional correlation function (**B**) of the PLLA/TAIC (60/40) blend after cold crystallization at the indicated temperatures.

In Figure 7A, showing the Lorentz-corrected SAXS profiles, the characteristic scattering vector is 0.32 nm^−1^ in the black curve (cold crystallization at 60 °C), corresponding to a long period of 19.6 nm. This value increases to 21.7 and 23.3 nm for temperatures of 80 °C and 100 °C respectively. According to the method introduced in Figure 3B, the thicknesses of crystal lamellae can be determined from Figure 7B. These are 6.9, 7.2, and 7.9 nm for cold crystallization at 60 °C, 80 °C, and 100 °C, respectively.

Long periods and thicknesses of crystal lamellae upon cold crystallization are both listed in Table 2. Crystallization at higher temperature produces higher long periods (*L*) and higher thicknesses of crystal lamellae (*d*). As a result, the crystallinities from SAXS (*X*_1_) show comparable magnitudes. The crystallinity from DSC (*X*_2_) increases with increasing crystallization temperature from 21.5% (60 °C) to 23.0% (100 °C). The crystallinity ratio between *X*_1_ and *X*_2_ therefore decreases from 1.64 (60 °C) to 1.50 (80 °C) and reaches 1.47 for the specimen upon cold crystallization at 100 °C. The decreasing magnitude of the crystallinity ratio suggests enhanced inclusion behaviors of TAIC during the cold crystallization of PLLA at higher temperatures.

**Figure 8:**
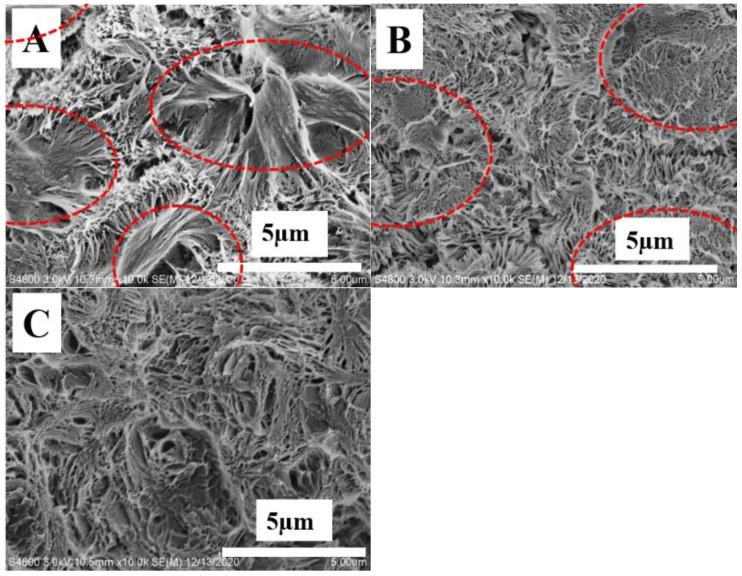
SEM images of PLLA/TAIC (60/40) blend upon etching with methanol on the specimen upon complete cold crystallization at 60 °C (**A**), 80 °C (**B**), and 100 °C (**C**).

The resultant porous structures upon etching with methanol (Figure 8) depend greatly on the temperature of cold crystallization. In Figure 8A, showing the SEM image of porous fracture surface from the specimen crystallized at 60 °C, some compact spherulites are obvious (red dash ellipses). The diameters of these are roughly several microns. In the regions among these spherulites, there are some porous structures, indicating the localization of TAIC in these regions before etching. This result makes it clear that most TAIC is located among PLLA spherulites. In other words, TAIC was expelled during the cold crystallization of PLLA at 60 °C. In Figure 8B, there can be seen two kinds of porous structures including smaller pores in spherulites (red dash ellipses) and larger pores among them. The occurrence of the former suggests that parts of were trapped in PLLA spherulites at 80 °C. Crystallization at 100 °C resulted in the disappearance of pores among spherulites (Figure 8C) and produced relatively “homogeneous” porous structures, corresponding to the enhanced inclusion behaviors of TAIC. The porous structures discussed above have good agreement with the evolution of crystallinity ratios (*X*_1_/*X*_2_) shown in Table 2.

**Figure 9:**
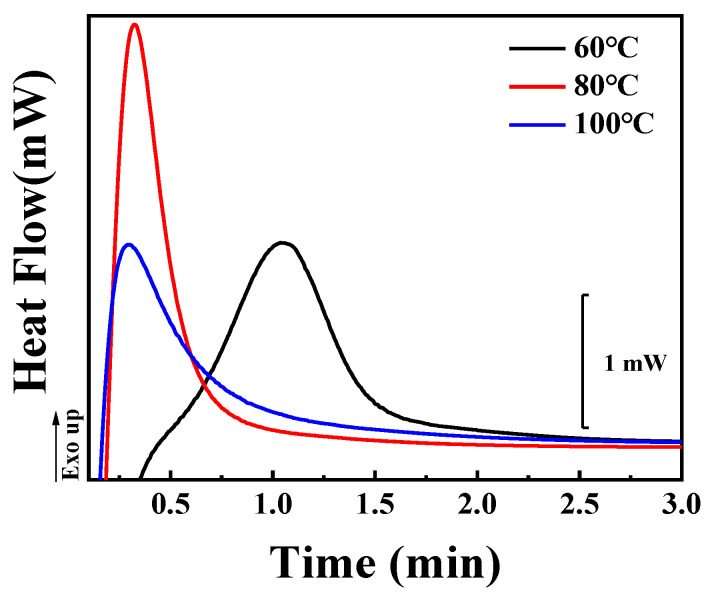
The kinetics of cold crystallization of the PLLA/TAIC (60/40) blend at the indicated temperatures.

In both inclusion and exclusion behaviors of TAIC during the crystallization of PLLA, the growth rate (*G*) of PLLA spherulites plays an important role. In the case of cold crystallization, however, it is impossible to track the growth of spherulites by means of POM because there are so many spherulites with smaller diameters (shown in Figure 8A) resulting from the high nucleation density at room temperature [41]. The kinetics of cold crystallization of the PLLA/TAIC blend was therefore investigated by means of DSC. As shown in Figure 9, it takes 2 min for PLLA to crystallize sufficiently at 60 °C. This period decreases to less than 1 min at 80 °C and at 100 °C. The higher crystallization temperature corresponds to the higher crystallization rate, which is the reason for the higher magnitude of growth rate of PLLA spherulites. As a result, more TAIC can be trapped, contributing to the enhanced inclusion behaviors, to the porous structures depicted in Figure 8, and to the lower magnitude of the crystallinity ratio (Table 2).

In the light of the discussion above, we can describe the inclusion/exclusion behaviors of TAIC during the crystallization of PLLA as follows: In the melting state, PLLA and TAIC are miscible. This conclusion is validated by the SEM images and DSC curves shown in Figure 1. During melt crystallization and cold crystallization, the localization of TAIC depends crucially on the crystallization conditions. Firstly, crystallization temperature plays an important role. A lower crystallization temperature produces lower magnitudes of growth rate of spherulites (Figure 5A–F and Figure 9). Consequently, more TAIC is expelled and then localizes in the regions among PLLA spherulites. This explains the higher crystallinity ratios (Table 1 and Table 2), the polyhedral boundaries from spherulites impingement (Figure 4D), and the porous structures among compact spherulites (Figure 8A). With higher crystallization temperatures, both the diffusion coefficient of TAIC and the growth rate of PLLA spherulites exhibit higher magnitudes. The increase in the latter, however, is much more dramatic than that of the former. In consequence, more and more TAIC is trapped in PLLA spherulites, accounting for the enhanced inclusion behaviors, the lower crystallinity ratios, and the absence of porous structures among PLLA spherulites (Figure 4C,F and Figure 8C). Secondly, melt crystallization and cold crystallization both exert significant influences on the inclusion and exclusion behaviors of TAIC. In the former, the specimen was cooled to a certain temperature directly from the melting state and sufficiently isothermally crystallized at this temperature. In this process, the spherulites exhibited much greater diameters (Figure 4A,D and Figure 5B) in comparison with cold crystallization (Figure 8A). Some TAIC was trapped in PLLA spherulites while some diffused to the growth front of PLLA crystals. Upon further crystallization of PLLA, more and more TAIC enrichment occurred in this region. Then, PLLA spherulites have no choice but to trap TAIC. During cold crystallization, a specimen previously stored in the refrigerator was heated to a certain temperature. The nucleation density, in this case, was much higher relative to melt crystallization, which is the reason for the smaller spherulites (Figure 8A). As a result, the volume fraction of the space among neighboring spherulites increases remarkably. There is enough space for TAIC to diffuse and enrich, accounting for the localization of TAIC in the regions among PLLA spherulites. Notably, there is an obvious difference in the localization of amorphous components between the polymer–small molecule blend and the polymer–polymer blend. In our previous work [27], the localization of PBSU was investigated during the crystallization of PVDF in our blend. The result indicated that the enrichment of PBSU in interspherulitic regions of PVDF took place when the blend underwent a two-phase regional reaction as shown in the phase diagram (i.e., a phase-separated state). In the results of Yan and his co-workers, poly(butylene adipate) with lower melting temperature localized in interspherulitic regions only when PBSU crystallized extremely slowly (two weeks) in their blend [9]. In this work, the diffusion coefficient of TAIC exhibits a much higher magnitude, corresponding to a stronger diffusion ability. As a result, it is more difficult for PLLA spherulites to trap TAIC, which is the reason for the enhanced exclusion behaviors relative to polymer–polymer blends.

## 4. Conclusions

Miscible PLLA/TAIC blend was taken as a model system to investigate the inclusion and exclusion behaviors of small molecules during the crystallization of polymers. In melt crystallization and cold crystallization, lower crystallization temperature results in enhanced exclusion behaviors of TAIC, which can be attributed to the lower growth rate of PLLA spherulites. With increasing crystallization temperature, the growth rate of PLLA spherulites exhibits a much higher magnitude, accounting for the higher fraction of included TAIC. Compared to melt crystallization, cold crystallization produces a much higher nucleation density and a resultant higher volume fraction of space among spherulites. This is the reason for the enrichment of TAIC in interspherulitic regions and for the enhanced exclusion of TAIC. Our results have significance not only for the basic understanding of crystallization in miscible blend systems containing crystalline polymers, but also for the performance improvement of polymer–additives blends.

## Figures and Tables

**Table 1:** Long period, thickness of lamellae, amorphous layer, crystallinity from SAXS and DSC upon melt crystallization at the indicated temperatures.

Temperature (°C)	*L* (nm)	*D* (nm)	AM (nm)	Crystallinity SAXS *X*_1_	Crystallinity DSC *X*_2_	*X*_1_/*X*_2_
60	18.9	6.9	12.1	36.5%	27.8%	1.31
80	20.3	7.3	13.0	36.0%	28.3%	1.27
100	21.2	7.5	13.7	35.4%	28.5%	1.24

**Table 2:** Long period, thickness of lamellae, amorphous layer, crystallinity from SAXS and DSC upon cold crystallization at the indicated temperatures.

Temperature (°C)	*L* (nm)	*d* (nm)	AM (nm)	Crystallinity SAXS *X*_1_	Crystallinity DSC *X*_2_	*X*_1_/*X*_2_
60	19.6	6.9	12.7	35.2%	21.5%	1.64
80	21.7	7.2	14.5	33.2%	22.1%	1.50
100	23.3	7.9	15.4	33.9%	23.0%	1.47

## Data Availability

The data presented in this study are available on request from the corresponding author.
